# Tool Health Monitoring of a Milling Process Using Acoustic Emissions and a ResNet Deep Learning Model

**DOI:** 10.3390/s23063084

**Published:** 2023-03-13

**Authors:** Mustajab Ahmed, Khurram Kamal, Tahir Abdul Hussain Ratlamwala, Ghulam Hussain, Mejdal Alqahtani, Mohammed Alkahtani, Moath Alatefi, Ayoub Alzabidi

**Affiliations:** 1 Department of Engineering Sciences, National University of Sciences and Technology, Islamabad 44000, Pakistan; 2 Mechanical Engineering Department, Faculty of Engineering, University of Bahrain, Isa Town 32038, Bahrain; 3 Industrial Engineering Department, College of Engineering, King Saud University, Riyadh 11421, Saudi Arabia

**Keywords:** acoustic emission, spectrograms, convolutional neural network, signal processing, feature extraction, tool health monitoring

## Abstract

In the industrial sector, tool health monitoring has taken on significant importance due to its ability to save labor costs, time, and waste. The approach used in this research uses spectrograms of airborne acoustic emission data and a convolutional neural network variation called the Residual Network to monitor the tool health of an end-milling machine. The dataset was created using three different types of cutting tools: new, moderately used, and worn out. For various cut depths, the acoustic emission signals generated by these tools were recorded. The cuts ranged from 1 mm to 3 mm in depth. In the experiment, two distinct kinds of wood—hardwood (Pine) and softwood (Himalayan Spruce)—were employed. For each example, 28 samples totaling 10 s were captured. The trained model’s prediction accuracy was evaluated using 710 samples, and the results showed an overall classification accuracy of 99.7%. The model’s total testing accuracy was 100% for classifying hardwood and 99.5% for classifying softwood.

## 1. Introduction

An important area of interest currently is intelligent manufacturing systems due to the fact that they can reduce downtime by using machine health to forecast early failure. Notably, in the woodworking sector, accurate tool health prediction is crucial and plays a significant role in reducing downtime and raising productivity. Monitoring the health of the tools used in an intelligent manufacturing system is one of the active research fields. At least one company loses more than 300 labor hours per year and more than USD 172 million as a result of financial penalties, lost income, idle staff time, and other factors, according to a survey of 72 large businesses [1]. Another study states that an hour lost due to downtime can incur losses ranging from USD 84 k to USD 108 k [2]. Additionally, it has been estimated that the annual cost to industries of unscheduled downtime might approach USD 50 billion, with machinery failure accounting for about half of that cost [3]. Machinery failure was rated as one of the top 10 leading causes of financial loss to industries in research that examined more than 11,000 company claims [4].

Predictive maintenance, which includes tool health monitoring, enables an industry to forecast the health of a tool and identify flaws such as dimensional inaccuracy, poor work piece surface roughness, excessive power consumption, etc. The gradual process of tool degradation might affect the caliber of the machining operation and result in a poor surface finish. When a tool makes contact with a work piece, a precise force is produced, and this force results in the generation of a number of signals in real time. The challenge is in developing a system that can develop an early warning system for failure by looking at the tool’s present state. A method to distinguish between TCMS is their mode of application, whether it is offline, online, or performing its operations in real time. A method is considered offline when the tool is removed from the jig for inspection or the machined parts are inspected for errors and then the process parameters are calibrated accordingly. The method in which the information is acquired directly (by measurements) or indirectly (by signals) but without stopping the machine or removing the tool is considered an online system [5]. A real-time system collects data continuously without hampering the operation of the machine and takes corrective actions with very little latency. More and more industries are shifting to real-time systems as they help in saving more money [6].

Various methodologies, such as vibration-based monitoring, acoustic signature analysis, sensor fusion-based techniques and so on, have been presented in this respect. M. Wang et al. [7] developed an approach based on the Continuous Hidden Markov Model (CHMM). The study developed a method for forecasting the RUL of milling cutters by watching cutting force signals, and it was discovered that CHMM is an incredibly useful instrument for tool condition monitoring since it just takes a limited number of training samples and minimal training time. F. J. Alonso et al. [8] addressed their study on the development of a system that employed vibration signals to analyze the characteristics of the tool. The vibration of the instrument was studied using cluster analysis and singular spectrum analysis (SSA). SSA is a spectrum estimation approach that makes no parameter assumptions. It decomposes the acquired signal into an extra set of time series and incorporates signal processing, multivariate geometry, multivariate statistics, dynamical systems, and classical time series analysis. In the tool, the flank wear estimates were estimated using a feed-forward backpropagation neural network. The study’s outcomes illustrate how rapid and reliable the recommended tool health monitoring method was. Research on a machine-learning-based method for troubleshooting face-milling tool equipment was presented by Madhusudana [9]. He employed histogram characteristics and the K-star algorithm for machine learning. Under various fault conditions, including with new and worn tools, the transmission of vibration signals was observed. The prominent traits that were extracted from each feature were sent to the classifier through a decision tree approach. The classification accuracy of the K-Star method was 94%, while that of the histogram features was 96%.

According to Sundaram et al. [10] study, vibration- or force-based tool monitoring approaches are inferior to AE-based tool monitoring systems. Throughout his investigation, he distinguished between two different emission types: a continuous emission and a burst emission that resulted in spikes in the time series graph of the signal. The goal of the investigation was to understand flank wear using AE signals. The tool’s wear was separated into three stages based on the passage of time: slow, moderate, and speedy. By observing the cumulative mean values of AE signals during these stages, it was possible to see the transition between time and cumulative AE parameters, such as cumulative average value, cumulative value of RMS, and cumulative area of mean. The study team discovered that Acoustic Emission Techniques (AET) are the best at detecting flank wear, according to the result. R. H. L. Da Silva et al. [11] investigated how to forecast a tool’s life throughout the milling process using cutting power data, acoustic emission signals, and probabilistic neural networks (PNN). At a frequency of 1.0 MHz, a piezoelectric sensor captured the AE signals. Examining the association between tool wear and AE signals, they were filtered out in the 100–230 kHz region. In this study, the combination of AE data with cutting power signals for tool wear prediction yielded a very promising result of 91% accuracy. P. Krishnakumar et al.’s study [12] examined the results of decision trees, SVM, naive bayes, and ANN among other ML techniques. The data required to classify the health of a tool were gathered using acoustic emission signals and vibration signals. While a titanium alloy was being cut in a high-speed precision machining facility, the signals were being gathered. SVM was shown to be superior to all other machine learning models with a reported accuracy of 99.26%, while ANN was found to be the second best algorithm taken into account in the study endeavor. C. Wang et al. [13] showed in their research how cutting ductile material makes the traditional approach of tool health monitoring utilizing AE continuous signals less accurate and more difficult. This is harder to do under Minimum Quantity Lubrication (MQL). This work provides a non-destructive approach using AE burst signals for analyzing tool condition under MQL to address this issue. The results showed that a linear fitting model can correctly identify the flank wear by using signals of acoustic emission caused by plastic deformation and material fracture. A relationship between AE burst signals and flank wear was created by grouping AE energy. As a result, a sophisticated and intricate system was created by all these signals. However, many academics have begun to pay attention to acoustic-based analysis. In 2017, B. Cuka and D.-W. Kim [14] developed a TCM for end milling based on fuzzy logic. Four different data signals were collected; they were cutting force signal, machining sound signal, spindle vibration signal, and spindle current signal.

Through the use of airborne acoustic emissions from an end-milling machine, this study provides a novel approach to an online indirect strategy for tool health monitoring. The proposed approach is a low-cost technique for tool health prediction as airborne acoustic emission is a contactless method, so there are no problems with sensor placement. A pre-trained convolutional neural network ResNet was used for the first time for monitoring tool health on two kind of wood using airborne acoustic emissions from an end-milling process. Varying depths of cut were used during the data acquisition phase to produce a disparate dataset for training and validation of the deep learning model. Table 1 shows the comparison of the proposed approach with previously carried out research work in this domain.

## 2. Proposed Technique

**Figure 1:**
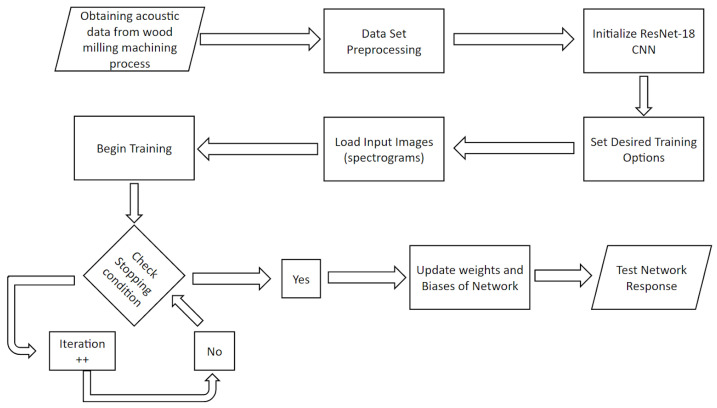
Flowchart of the proposed technique.

The comprehensive flowchart of the suggested technique is shown in Figure 1. First, a regular microphone was used to record the AE signals generated during the machining process. After that, preprocessing is used to turn the raw AE signals into two-dimensional images. Later, a ResNet-18 architecture with various layers was created. The images were fed to the architecture to start the training process after the parameters and hyperparameters were set up in accordance with the suggested design requirement. After each training, the classification results were validated, and the algorithm’s performance was assessed.

## 3. Materials and Methods

### 3.1. Convolutional Neural Networks

**Figure 2:**
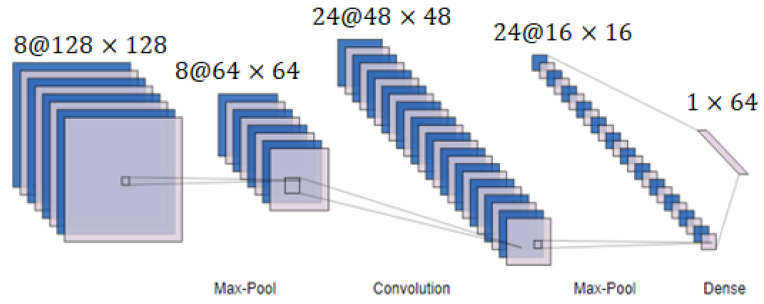
Architecture of a CNN.

Convolutional neural networks are widely used in the field of computer vision (CNN or convnets). An ANN only has a dense layer, but a CNN has extra convolution layers and pooling layers. This is the main distinction between an ANN and a CNN. Here, Figure 2 is displayed to illustrate the fundamental layout of a CNN. To address complicated problems and improve performance, deeper convolution networks frequently repeat layers of convolution and pooling.

A convolutional layer serves as the basis of a CNN, and it is from the name of this layer that the term CNN is formed. This layer’s primary function is to compute the kernel dot product from the input, and then output the result as an activation map or feature map. Learnable parameters that extract high-level data from the input are known as kernels, which is a filter type. Inferring that the kernel updates itself after each iteration via backpropagation is what we mean by a learnable parameter. The pooling layer performs a similar operation as the convolution layer, but a limitation of the convolution layer is addressed in the pooling layer. The limitation is that the exact position of the features is stored in the feature map, which means any slight change in the input will require a different feature map. This limitation is addressed by down-sampling, in which key features of input are kept with low resolution. This process of down-sampling is performed by a pooling layer. Following the activation function, the pooling layer is introduced. There are various pooling layer types, including maximum, average, and global pooling. Almost all ANN types feature a layer known as the dense layer. There is a connection between each neuron in the layer above and below it. This layer is essential for categorizing data. Other terms for this layer are deeply connected layer and fully connected layer.

### 3.2. Residual Neural Networks

ResNets are built using a modified version of the CNN architecture to address the issue of “vanishing or exploding gradients” in extremely deep convents, where a very small gradient prevents the updating of weights. This issue causes the training error to start rising rather than falling. The addition of residual blocks to the design provided a solution to this issue. Residual blocks create a proper link between each layer, but they also create a shortcut (a skip connection) that allows us to move activation from a shallow layer straight into a much deeper layer. A ResNet is created by stacking these residual blocks sequentially. There are several leftover blocks in ResNet. The below equation demonstrates a ResNet block.

*y* = *f*(*x*) + *x* (1)

where *x* is the input to the residual block and *f*(*x*) is the output after various operations have been applied. As a result, the output of the residual block is *f*(*x*) + *x*, which represents the propagation of information from the previous layer to the subsequent layer. The batch normalization and ReLU layers come after the convolutional layers in a residual block.

In deep learning, data are preprocessed and usually normalized to make it follow the normal distribution to avoid untimely saturation of activation functions. However, the issue of internal covariate shift still arises in the intermediate layers because of constantly varying distribution after activation. The batch normalization layer is inserted after the activation layer to normalize the output of each layer, which is going to become the input for another layer. Mean and variance are calculated, and the formerly computed batch statistics are used to normalize the input.

**Figure 3:**
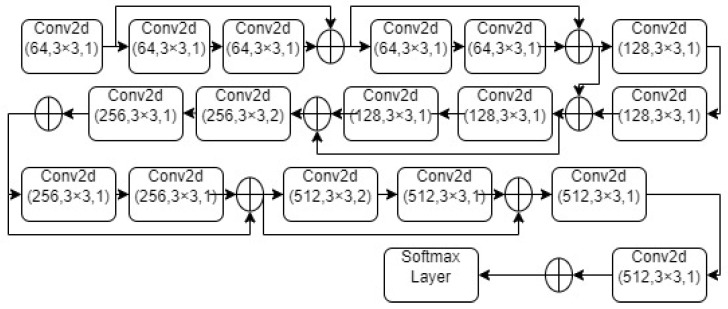
Architecture of ResNet-18. Values in brackets represent the number of filter present in the layer, the size of the filter, and the stride of the filter.

The ResNet-18 nomenclature refers to the 17 convolutional layers and one fully connected layer that make up the architecture chosen for this work. Eight residual blocks are produced by 16 layers of convolution. A layer of activation and a layer of batch normalization follow each layer of convolution. Following the first residual block and following the last residual block that links to the dense layer, respectively, are two pooling layers. The architecture of an 18 layer Residual Neural Network is shown in Figure 3.

### 3.3. Experimental Procedures and Setup

**Figure 4:**
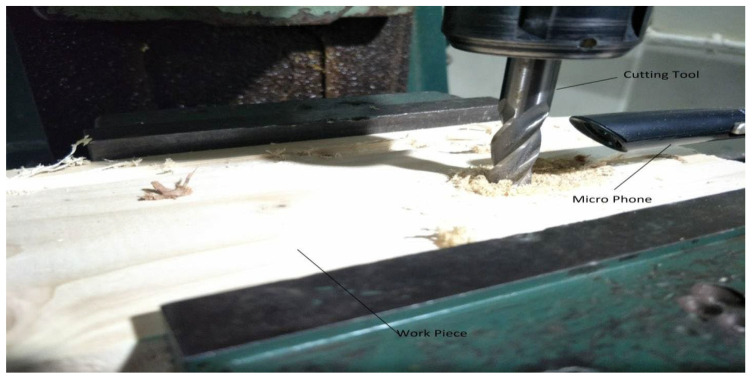
Experimental setup.

To obtain AE signals, the end-milling machine model PMTFK4 was employed. In the studies, two distinct types of woods were sliced using three different cutting tools under various settings as shown in Figure 4.

**Figure 5:**
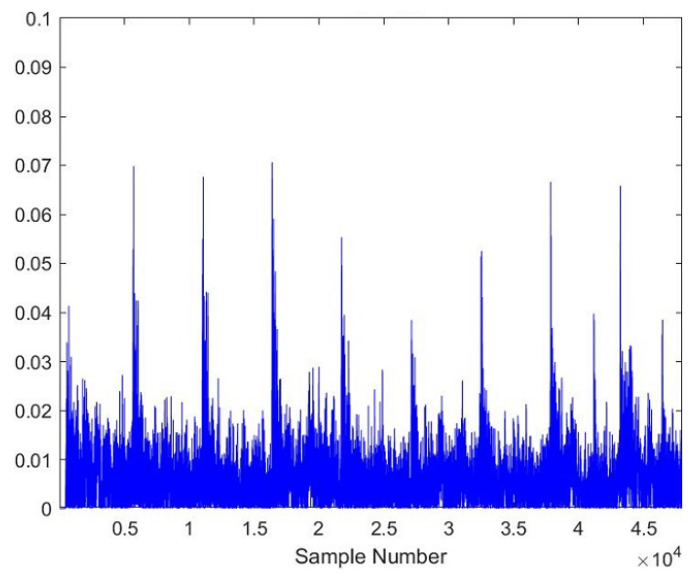
RMS plot of Hardwood, Average Tool, 3 mm depth-of-cut.

**Figure 6:**
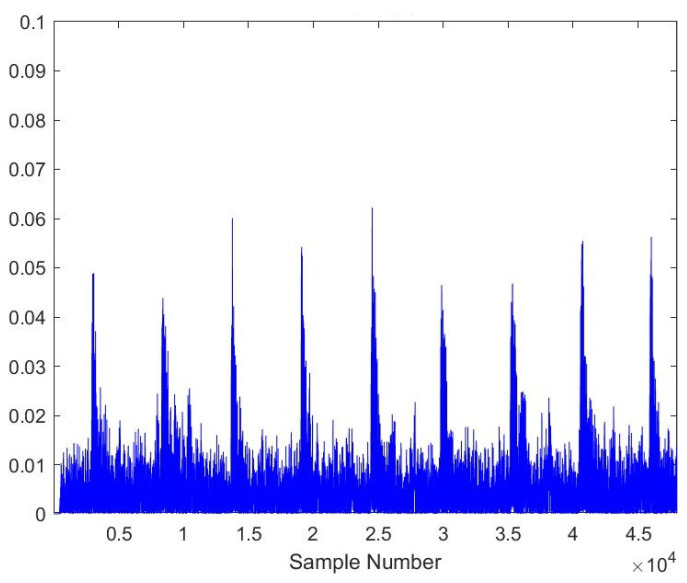
RMS plot of Hardwood, New Tool, 1 mm depth-of-cut.

**Figure 7:**
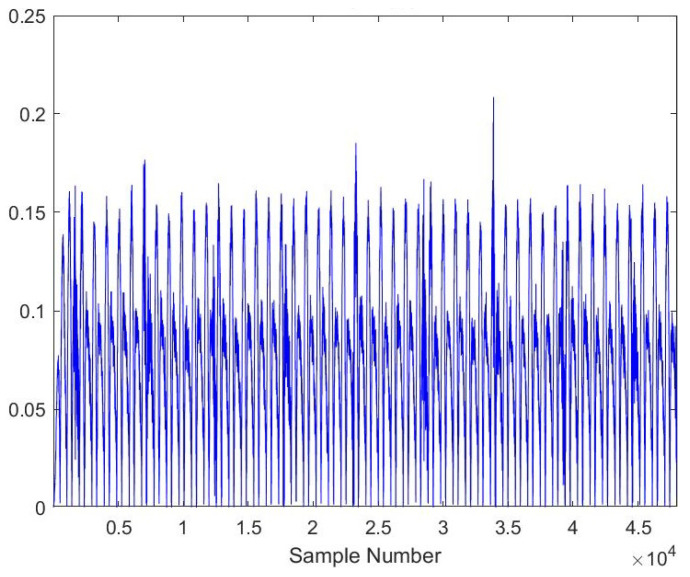
RMS plot of Hardwood, Worn-out Tool, 2 mm depth-of-cut.

**Figure 8:**
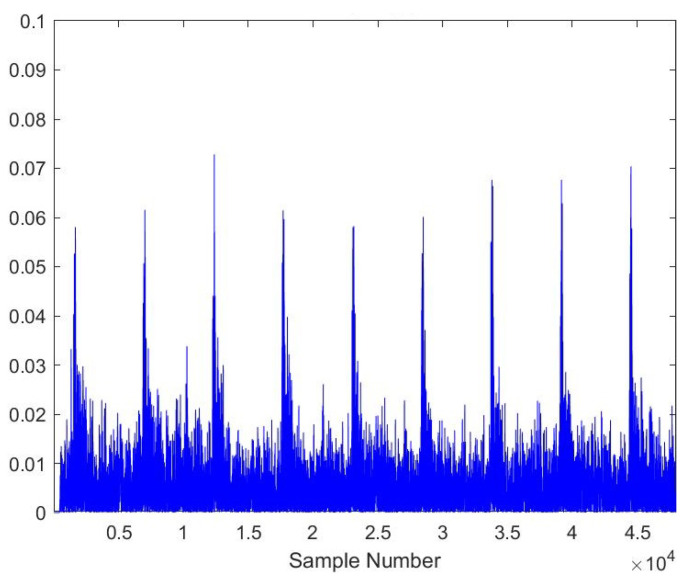
RMS plot of Softwood, Average Tool, 2 mm depth-of-cut.

**Figure 9:**
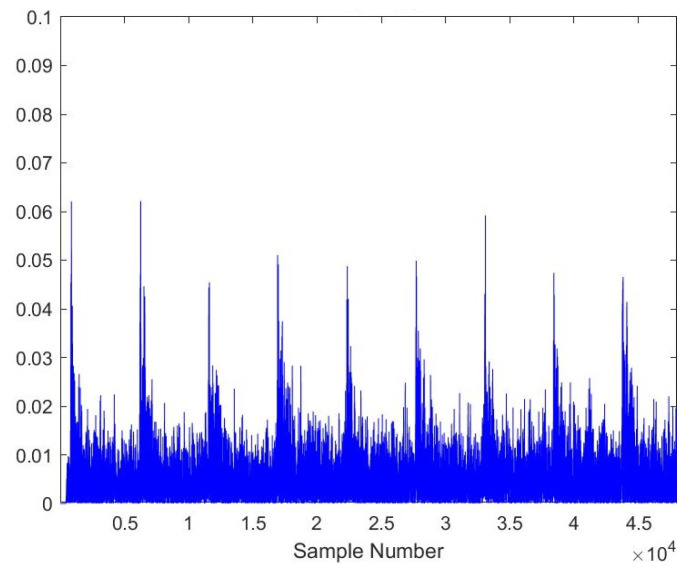
RMS plot of Softwood, New Tool, 1 mm depth-of-cut.

**Figure 10:**
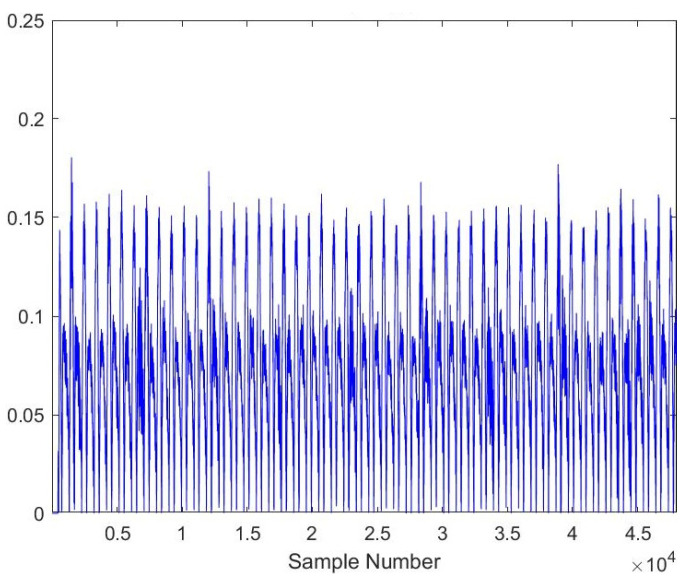
RMS plot of Softwood, Worn-out Tool, 3 mm depth-of-cut.

As the AE signals lie in the audible frequency range of 20 Hz to 20 kHz, a cheap microphone was used to record the acoustic emission signals at a feed rate of 21.5 mpm. The sampling frequency of the microphone was set to 44.1 kHz to meet the requirements of the Nyquist criteria. A total of 28 samples, each lasting 10 s, were recorded. The recorded samples were broken down into one-second-long samples to gain RMS plots of the signals. A total of more than 5040 samples were obtained of one second length, out of which 710 samples were kept for testing the model. Figure 5, Figure 6, Figure 7, Figure 8, Figure 9 and Figure 10 represent the RMS values of single second-long AE signals. To capture the most acoustic emissions and minimize background noise, the microphone was placed quite close to the tool. During data collection, the spindle was turning at a 45 RPM speed. The AE signals were captured at three distinct depths of cut, namely 1 mm, 2 mm, and 3 mm.

The experiments are performed for two types of wood: hard wood (Pine) and soft wood (Himalayan Spruce). Different physical properties of the woods are presented in Table 2, which shows the wood-related information.

Moreover, the architecture used in this research is ResNet, short for Residual Network, which is a version of CNN. The ResNet-18 moniker comes from the fact that the architecture chosen for this work has one fully connected layer and 17 convolutional layers. Convolution across 16 layers yields 8 residual blocks. An activation and a batch normalization layer come after each convolutional layer. After the first residual block and after the last residual block that links to the dense layer, there are two pooling layers present.

Table 3 below gives more information about the filters in the architecture.

## 4. Results

### Spectrograms

In order to create spectrograms, the 10 s input AE signals are shortened to 1 s. A spectrogram is a three-dimensional representation of a signal that plots time on the horizontal x-axis, frequency on the vertical y-axis, and the energy of the spectrum at a given moment as indicated by colors. The spectrograms that were created under various cutting conditions are discussed below.

**Figure 11:**
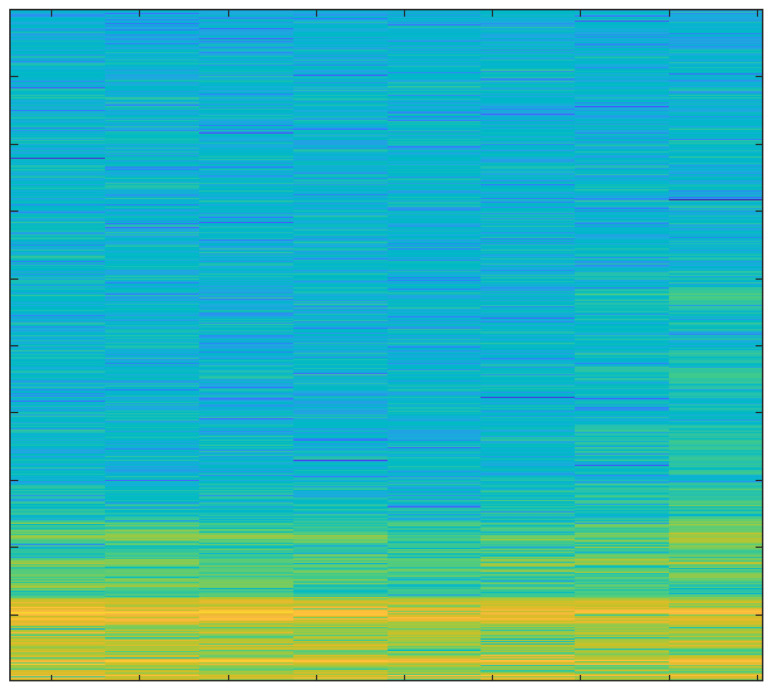
Hardwood, Average Tool, 3 mm depth-of-cut.

The spectrogram in Figure 11 represents both low and high energies as an average tool produces more sound than a new one.

**Figure 12:**
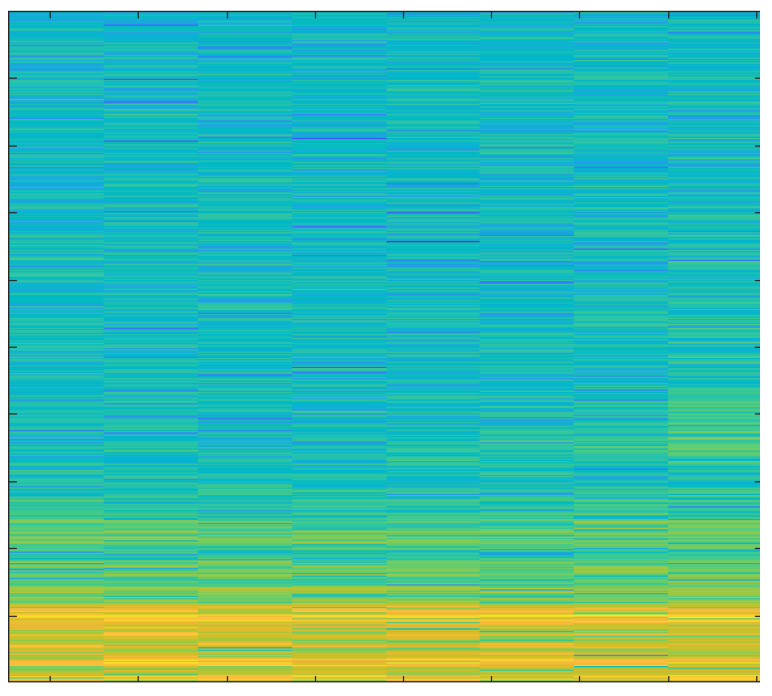
Hardwood, New Tool, 1 mm depth-of-cut.

The spectrogram in Figure 12 displays reduced power, since the noise generated by the new tool is extremely minimal. 

**Figure 13:**
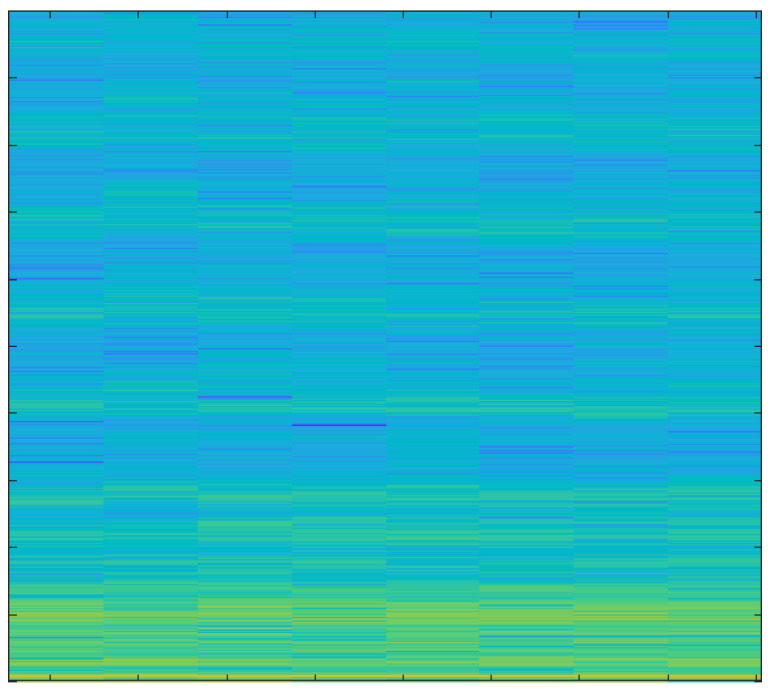
Hardwood, Worn-out Tool, 2 mm depth-of-cut.

The spectrogram in Figure 13 shows very high energy values because of the worn-out tool.

**Figure 14:**
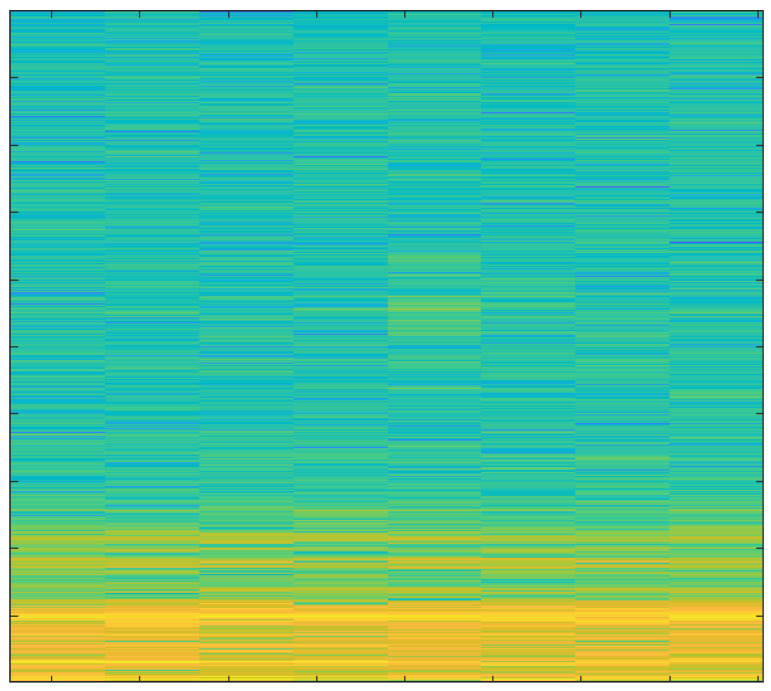
Softwood, New Tool, 1 mm depth-of-cut.

The spectrogram in Figure 14 shows even lower energy values compared to the spectrogram of hardwood milling with a new tool; that is because lower noise is generated when milling softwood.

**Figure 15:**
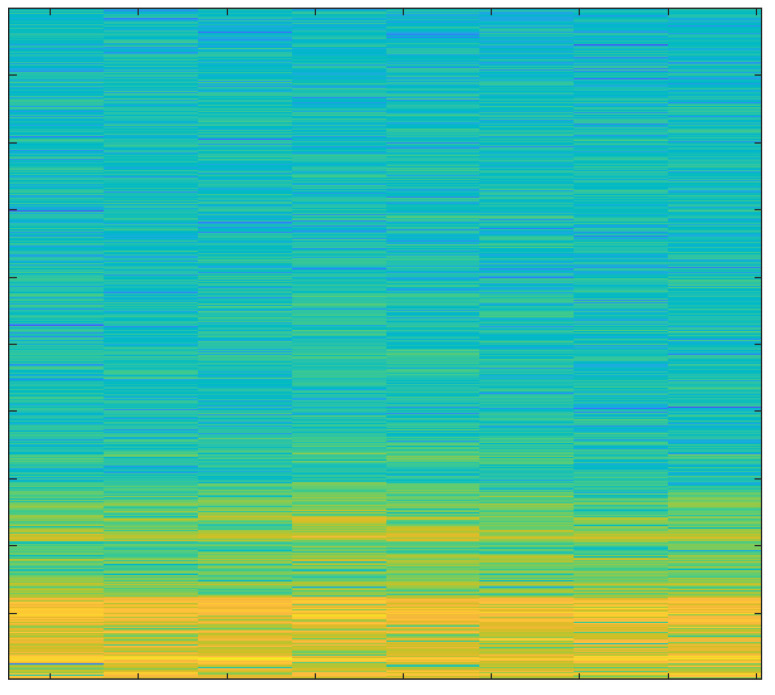
Softwood, Average Tool, 3 mm depth-of-cut.

The spectrogram in Figure 15 shows that high energies are dominant. It was observed that energy level increased substantially when depth-of-cut was increased while working with a moderately used tool.

**Figure 16:**
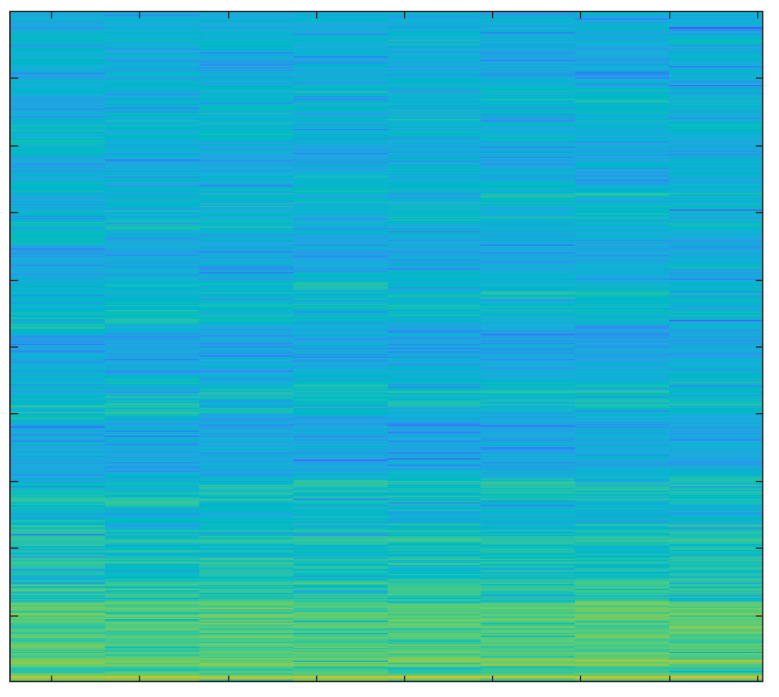
Softwood, Worn-out Tool, 2 mm depth-of-cut.

The spectrogram in Figure 16 shows very high energies because of the blunt edges of the tool.

The primary focus of this study was the use of spectrograms to analyze the acoustic emission (AE) signals obtained from an end-milling machine. Spectrograms are a powerful tool for visualizing the frequency content of a signal over time and can provide valuable information about the health of a tool.

In our study, we employed a deep learning approach to monitor the health of a tool using AE signals. By breaking the signal into overlapping segments and plotting the frequency content of each segment, a spectrogram can be created that shows how the frequency content of the signal changes over time. This technique allows for a more detailed representation of the signal’s frequency content over a shorter period of time.

Our analysis of the spectrograms revealed a number of patterns and trends in the frequency content of the AE signals that were associated with different stages of tool wear. For example, we observed an increase in high frequency content in the AE signals as the tool wear progressed. This finding is consistent with previous research in the field and suggests that our deep learning approach is effective for monitoring the health of a tool using AE signals.

In summary, the results of this study demonstrate the utility of spectrograms as a primary tool for monitoring the health of a tool using AE signals. By analyzing the frequency content of the AE signals over time, we were able to identify patterns and trends that were associated with different stages of tool wear. Additionally, by using a deep learning approach, we were able to extract important features from the signals and analyze those using spectrograms which further strengthen our findings and results. This information can be used to develop more effective strategies for tool maintenance and prolong the tool’s lifespan.

## 5. Discussion

As discussed earlier, the pre-trained ResNet convolutional neural network algorithm is used to train the network. By assessing the model’s accuracy, the architecture of the suggested model, and comparing the results with earlier research, the results are obtained. The model underwent 136 iterations of training on five epochs of training data. With two exceptions, the model’s accuracy was 100% in every situation. In those two cases, when working on softwood with depth-of-cuts of 3 mm and 1 mm, respectively, the model was unable to adequately differentiate between an average tool and a worn-out tool. The model’s accuracy was 84.9% when trained on softwood, 1 mm, worn-out tool and softwood, 3 mm, average tool. The categories softwood, 1 mm, worn-out tool and softwood, 3 mm, average tool were tested with 99.7% accuracy using the model. The calculation showed that the accuracy was nearly always 100%. On softwood, 3 mm, average tool, the model’s accuracy throughout training was 99%. The model’s testing accuracy for softwood, 3 mm, and average tool was 95.6%.

**Figure 17:**
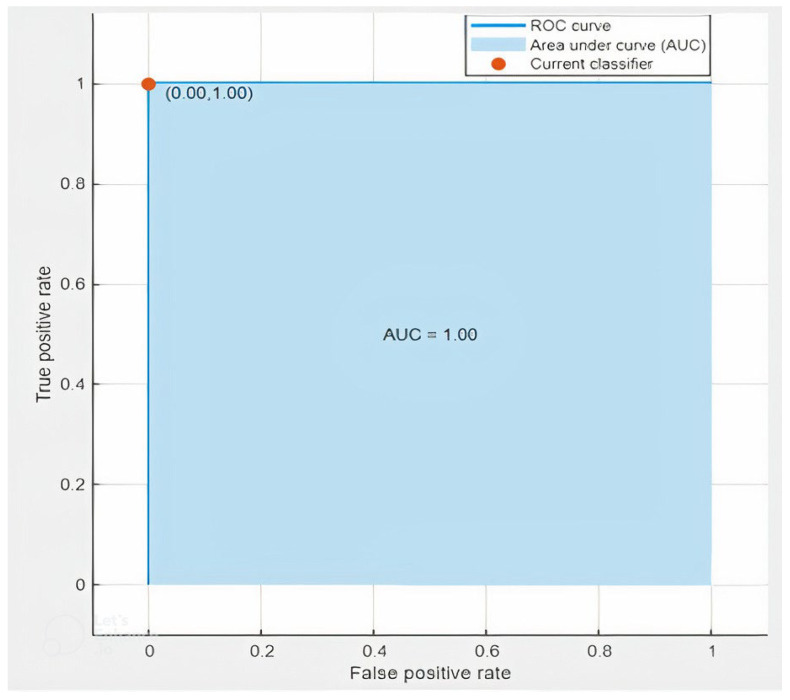
ROC Curve of Hardwood, Worn-out Tool, 1 mm.

**Figure 18:**
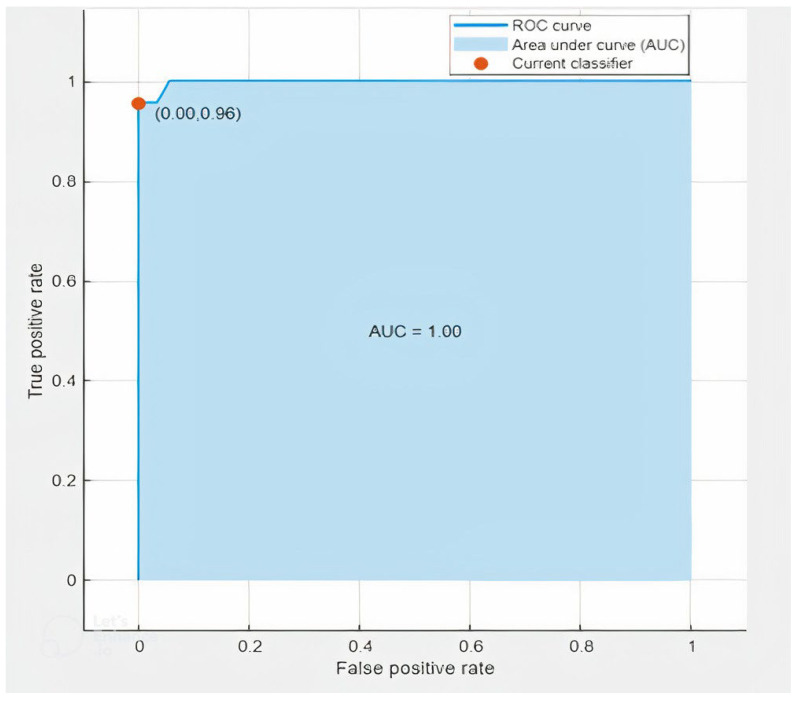
ROC Curve of Softwood, Average Tool, 3 mm.

The ROC curve shows how well a classification model performs across all classification levels. The ROC graph shows the true positive rate and the false positive rate, two parameters. To calculate the points on a ROC curve, we might evaluate a logistic regression model more than once with various classification criteria, but this would be wasteful. Thankfully, we have a quick, sorting-based method called AUC that can provide these data. Area under the ROC Curve is referred to as AUC. AUC quantifies the full two-dimensional area beneath the entire ROC curve, from (0, 0) to (1, 1), in other words (1, 1) (1, 1). We created two ROC charts for this investigation, which are displayed below. Figure 17 shows the ROC curve for the 1 mm worn-out tool case on hardwood, whereas Figure 18 shows the plot for the 3 mm average tool on softwood.

The performance parameters such as recall, accuracy, precision, and F-score are shown in the Table 4 below for each example which can be described in the form of equations as follows:

*Accuracy* (%) = (*TP* + *TN*)/(*TP* + *TN* + *FN* + *FP*) × 100 (2)

*Precision* (%) = *TP*/(*TP* + *FP*) × 100 (3)

*Recall* (%) = *TP*/(*TP* + *FN*) × 100 (4)

*F-Score* (%) = (2 × (*Recall* × *Precision*)/(*Recall* + *Precision*)) × 100 (5)

where *TP*, *TN*, *FP*, and *FN* stand for true positive, true negative, false positive, and false negative, respectively. A dataset was prepared to be assigned into the training and testing dataset type in order to access these parameters. The remaining 30% of the dataset was set aside for testing and validation, with 70% designated for training. The next step was fine-tuning the architecture with various convolution filter sizes, batch normalization layers, and the number of filters. The number of epochs for training was set to five with 136 iterations, which was achieving a consistent and accurate classification.

Most of the time, the model’s recall was assessed to be 100%. The model had an 82.9% recall rate when trained on softwood, 1 mm, worn-out tool. When evaluating the model for softwood, 1 mm, worn-out tool, the recall score was 94.8%. The model’s F-score in this instance was 90.6% when trained on softwood, 1 mm, worn-out tool, and 84.9% when trained on softwood, 3 mm, and average tool. While testing the model, the F-score for softwood 1 mm, worn-out tool” was 97.3%, and for softwood 3 mm, average tool, it was 97.7%.

**Figure 19:**
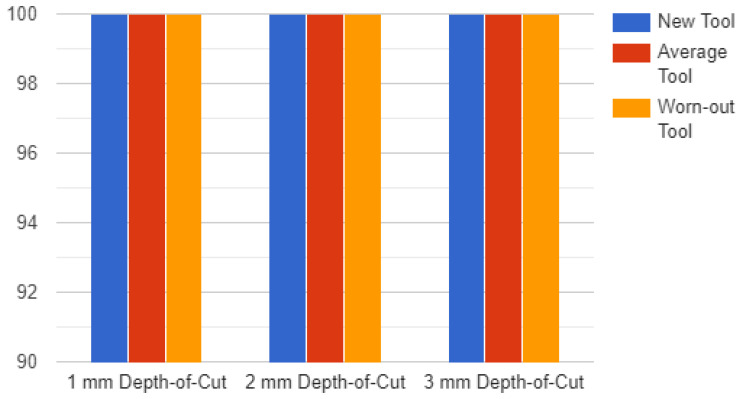
Accuracy Chart for Hardwood.

**Figure 20:**
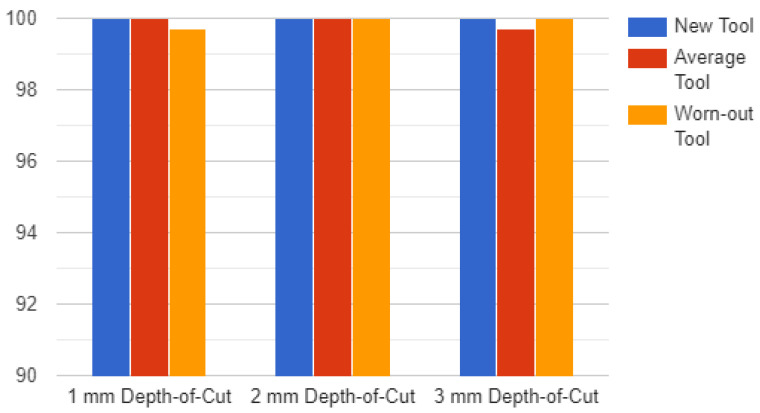
Accuracy Chart for Softwood.

Figure 19 shows the accuracy of the model for hardwood and Figure 20 shows the accuracy of the model for softwood.

The findings of the recommended strategy performed better than the most recent sophisticated techniques covered in the literature. Madhusudana [9] presented his study on a machine-learning-based method for identifying faults in face-milling machines. The K-Star method had a classification accuracy of 94%, and histogram features had a classification accuracy of 96%. Moreover, using probabilistic neural networks (PNN), cutting power data, and acoustic emission signals, R. H. L. Da Silva et al. [11] provided a study that analyzed the prediction of the life of a tool in the milling process. His research on using cutting power and AE signals together to estimate tool wear produced an excellent result of 91% accuracy. Our experimental results showed an overall classification accuracy of 99.7%. The model’s total testing accuracy was 100% for classifying hardwood and 99.5% for classifying softwood. When comparing the results to previous research, it was found that the proposed method performed better than other sophisticated techniques. The performance of the model was evaluated using ROC curves, AUC, and performance metrics such as recall, accuracy, and F-score. This study provides a new approach to identifying the wear state of cutting tools using AE signals and demonstrates the potential for this method to be used in industrial settings. However, further research is needed to improve the model’s ability to differentiate between average and worn-out tools in certain cases.

One of the primary advantages of this research is the use of spectrograms to analyze AE signals. Spectrograms are a powerful tool for visualizing the frequency content of a signal over time, and can provide valuable information about the health of a tool. By breaking the signal into overlapping segments and plotting the frequency content of each segment, a spectrogram can be created that shows how the frequency content of the signal changes over time. This technique allows for a more detailed representation of the signal’s frequency content over a shorter period of time, which is important for accurate monitoring of the health of a tool.

Another benefit of this project is the use of a deep learning approach to monitor the health of a tool using AE signals. The deep learning approach, specifically the ResNet convolutional neural network algorithm, was effective in extracting important features from the signals and analyzing those using spectrograms. This approach can be used to develop more effective strategies for tool maintenance and to prolong the tool’s lifespan, which can result in cost savings for manufacturers and industries that rely on end-milling machines. Furthermore, the use of ROC curves and AUC as performance metrics provides a comprehensive understanding of the model’s performance. ROC curves are widely used to evaluate the performance of binary classifiers, and AUC is a widely used measure of classification performance. These metrics provide a quantitative measure of the model’s performance, which helps to validate the results of this study.

However, with the benefits it is important to look at the limitations of the research. The main limitation of this study is the limited generalizability of the results. The study only used two types of wood, pine and Himalayan spruce, which limits the generalizability of the results to other types of materials. Additionally, the study only used one end-milling machine model, the PMTFK4, which limits the generalizability of the results to other types of machines. Furthermore, the study only used one type of cutting tool, which limits the generalizability of the results to other types of tools. These limitations mean that the results of this study cannot be directly applied to other types of materials, machines, and tools.

Another limitation is the model’s accuracy was lower when working on softwood with depth-of-cuts of 3 mm and 1 mm, which may indicate that the model may not perform well on other types of materials. This could be due to the different physical properties of softwood compared to hardwood, and further research is needed to understand how these properties affect the performance of the model. Despite these limitations, the results of this study provide valuable insights into the use of spectrograms and deep learning for monitoring the health of a tool using AE signals, and further research is needed to further understand the performance of the model and how it can be applied to other types of materials, machines, and tools.

In summary, the use of spectrograms and the deep learning approach, specifically the ResNet convolutional neural network algorithm, was found to be effective in extracting important features from the signals and analyzing those using spectrograms, which can lead to more effective strategies for tool maintenance and prolonging the tool’s lifespan. The use of ROC curves and AUC as performance metrics also provided a comprehensive understanding of the model’s performance. However, the main limitation of this study is the limited generalizability of the results as it only used two types of wood, one end-milling machine model, and one type of cutting tool.

## 6. Conclusions

Manufacturing is a key component of modern economies, and much research is conducted in this field to ensure its continued success. Tool health monitoring is one such method for lowering machine downtime and the expenses associated with it. This research examined the literature of methods used in various sectors to assess the health of tools. The experimental method for this study comprises the collection of unprocessed AE signals produced close to the tool. Different tool classes, including new, moderately used, and worn-out tools, were employed to produce a range of acoustic emissions. The depth-of-cut, which ranged from 1 mm to 3 mm, was another parameter that was changed throughout the capture of the signal. The model’s testing accuracy, which was evaluated on 15% of the dataset, was 99.7%, while the training accuracy was 99%. The model was able to identify between every class properly, with the exception of softwood, 3 mm, average tool and softwood 1 mm, worn-out tool, although even for those classes, the proportion of wrong classification was less than 1%. The suggested model performed incredibly well when compared to previously used methods. Additionally, the following points might be taken into consideration for the future:

- The proposed model can be enhanced further by gathering more relevant data from the industry and training the model on that data to develop a comprehensive system according to industry needs.
- The training time of the model can be further reduced by incorporating computers with high-end GPUs which are used for training deep learning models.
- A better-quality microphone can be incorporated which is able to catch more ranges of frequency and has the built-in feature of active noise cancellation.
- Comprehensive understanding of the tool’s health can be considered.
- Testing the model on a wider variety of machines and tools to increase its generalizability to different industrial settings.
- Investigating the use of other machine learning algorithms and techniques, such as deep reinforcement learning, to improve the model’s accuracy and performance.
- Developing a real-time monitoring system that can be integrated into industrial processes to provide real-time notifications of tool wear.
- Conducting further research to investigate the potential of using AE signals for monitoring other types of machinery and equipment in industrial settings.
- Developing a cost–benefit analysis to determine the financial advantages of implementing this method in industrial settings.
- Conducting case studies with industrial partners to validate the proposed method in real-world scenarios.
- Developing a user-friendly interface to make it easier for operators to interpret the results and take appropriate actions.
- Investigate the possibility of incorporating other parameters to improve the model’s ability to identify the wear state of the tools.

## Figures and Tables

**Table 1:** Comparison of the proposed approach with previously carried out research.

Researcher	Approach	Application	Accuracy
Madhusudana [9]	K-star Algorithm	Fault diagnosis of tools in face milling	96%
R. H. L. Da Silva et al. [11]	Probabilistic Neural Network	Tool life prediction using AE and cutting power signals	91%
P. Krishnakumar et al. [12]	Support Vector Machines	High-speed precision machining of a titanium alloy	99.26%
M. Arsalan et al. [15]	Convolutional Neural Network	Tool life prediction while working on Teflon, mild steel, and aluminum workpieces	99%
S. Rangwala and D. Dornfeld [16]	Artificial Neural Network	Tool health prediction using noisy signals	95%
Proposed Approach	Residual Neural Network	Wood-milling machining process on hard and soft wood	100% (hardwood) 99.5% (softwood)

**Table 2:** Wood-related data.

Tree Name	Biological Name	Specific Gravity	Modulus of Rupture	Density
Pine (Hard)	Pinus	0.40	64.1 MPa	430–570 kg/m^3^
Himalayan Spruce (Soft)	Picea Smithiana	0.39	39 MPa	333–365 kg/m^3^

**Table 3:** Filter data on layer of convolution.

Residual Block Number	Size of Filter	Number of Filters	Number of Convolutional Layers
1	3 × 3	64	1
2	3 × 3	64	4
3	3 × 3	128	4
4	3 × 3	256	4
5	3 × 3	512	4

**Table 4:** Performance metrics for tool health monitoring.

Work Pieces Types	Performance Parameter	New Tool	Average Tool	Worn-Out Tool	Average
Hardwood, 1 mm depth-of-cut	Accuracy	100%	100%	100%	100%
	Precision	100%	100%	100%	100%
	Recall	100%	100%	100%	100%
	F-score	100%	100%	100%	100%
Hardwood, 2 mm depth-of-cut	Accuracy	100%	100%	100%	100%
	Precision	100%	100%	100%	100%
	Recall	100%	100%	100%	100%
	F-score	100%	100%	100%	100%
Hardwood, 3 mm depth-of-cut	Accuracy	100%	100%	100%	100%
	Precision	100%	100%	100%	100%
	Recall	100%	100%	100%	100%
	F-score	100%	100%	100%	100%
Softwood, 1 mm depth-of-cut	Accuracy	100%	100%	99.7%	99.9%
	Precision	100%	100%	100%	100%
	Recall	100%	100%	94.8%	98.2%
	F-score	100%	100%	97.3%	99.1%
Softwood, 2 mm depth-of-cut	Accuracy	100%	100%	100%	100%
	Precision	100%	100%	100%	100%
	Recall	100%	100%	100%	100%
	F-score	100%	100%	100%	100%
Softwood, 3 mm depth-of-cut	Accuracy	100%	99.7%	100%	99.9%
	Precision	100%	95.6%	100%	98.5%
	Recall	100%	100%	100%	100%
	F-score	100%	97.7%	100%	99.2%

## Data Availability

The data presented in this study are available on request from the corresponding author.

## References

[1] ISA Interchange (2022). World’s Largest Manufacturers Lose $1 Trillion/Year to Machine Failure. https://blog.isa.org/worlds-largest-manufacturers-lose-1-trillion/year-to-machine-failure..

[2] McHatton D. (2017). 10 Ways Preventative Maintenance Can Assist in Reducing Downtime. https://www.sageautomation.com/blog/10-ways-preventative-maintenance-can-assist-in-reducing-downtime.

[3] (2022). Unlocking Performance How Manufacturers Can Achieve Top Quartile Performance. https://partners.wsj.com/emerson/unlocking-performance/how-manufacturers-can-achieve-top-quartile-performance/.

[4] (2018). Top Ten Causes of Financial Loss for Businesses. https://www.sherwininsurance.co.uk/2018/07/20/top-ten-causes-of-financial-loss-for-businesses/.

[5] Nath C. (2020). Integrated Tool Condition Monitoring Systems and Their Applications: A Comprehensive Review. Procedia Manuf..

[6] Mohamed A., Hassan M., M’Saoubi R., Attia H. (2022). Tool Condition Monitoring for High-Performance Machining Systems—A Review. Sensors.

[7] Wang M., Wang J. (2012). CHMM for tool condition monitoring and remaining useful life prediction. Int. J. Adv. Manuf. Technol..

[8] Alonso F., Salgado D. (2008). Analysis of the structure of vibration signals for tool wear detection. Mech. Syst. Signal. Process.

[9] Madhusudana C.K., Kumar H., Narendranath S. (2016). Condition Monitoring of face milling tool using K-star algorithm and histogram features of vibration signal. Eng. Sci. Technol. Int. J..

[10] Sundaram S., Senthilkumar P., Kumaravel A., Manoharan N. (2008). Study of flank wear in single point cutting tool using acoustic emission sensor techniques. ARPN J. Eng. Appl. Sci..

[11] Da Silva R.H.L., da Silva M.B., Hassui A. (2016). A probabilistic neural network applied in monitoring tool wear in the end milling operation via acoustic emission and cutting power signals. Mach. Sci. Technol..

[12] Krishnakumar P., Rameshkumar K., Ramachandran K.I. (2018). Machine learning based tool condition classification using acoustic emission and vibration data in high speed milling process using wavelet features. Intel. Decis. Technol..

[13] Wang C., Bao Z., Zhang P., Ming W., Chen M. (2019). Tool wear evaluation under minimum quantity lubrication by clustering energy of acoustic emission burst signals. Measurement.

[14] Cuka B., Kim D.-W. (2017). Fuzzy logic based tool condition monitoring for end-milling. Robot. Comput. Integr. Manuf..

[15] Arslan M., Kamal K., Sheikh M.F., Khan M.A., Ratlamwala T.A.H., Hussain G., Alkahtani M. (2021). Tool Health Monitoring Using Airborne Acoustic Emission and Convolutional Neural Networks: A Deep Learning Approach. Appl. Sci..

[16] Rangwala S., Dornfeld D. (1990). Sensor integration using neural networks for intelligent tool condition monitoring. J. Eng. Ind..

